# Mechanistic role of long non-coding RNAs in the pathogenesis of metabolic dysfunction-associated steatotic liver disease and fibrosis

**DOI:** 10.1136/egastro-2024-100115

**Published:** 2024-11-18

**Authors:** Henry Wade, Kaichao Pan, Bingrui Zhang, Wenhua Zheng, Qiaozhu Su

**Affiliations:** 1 School of Biological Sciences, Queen’s University Belfast, Belfast, UK; 2 Endocrinology Group, Advocate Illinois Masonic Medical Center, Chicago, Illinois, USA; 3 Faculty of Health Science, University of Macau, Macau, China

## Abstract

Metabolic dysfunction-associated steatotic liver disease (MASLD), previously referred to as non-alcoholic fatty liver disease, encompasses a broad range of hepatic metabolic disorders primarily characterised by the disruption of hepatic lipid metabolism, hepatic lipid accumulation and steatosis. Severe cases of MASLD might progress to metabolic dysfunction-associated steatohepatitis, characterised by hepatic inflammation, hepatocyte ballooning degeneration, activation of hepatic stellate cells (HSCs) and fibrogenesis. It may further progress to hepatocellular carcinoma. In the liver, long non-coding RNAs (lncRNAs) target multiple metabolic pathways in hepatocytes, HSCs, and Kupffer cells at different stages of MASLD and liver fibrosis. In this study, we overview recent findings on the potential role of lncRNAs in the pathogenesis of MASLD and liver fibrosis via modulation of de novo lipid synthesis, fatty acid β-oxidation, lipotoxicity, oxidative stress, metabolic inflammation, mammalian target of rapamycin signalling, apoptosis, ubiquitination and fibrogenesis. We critically assess the literature reports that investigate the complex interplay between lncRNA, microRNA and key mediators in liver injury, in both human participants and animal models of MASLD and liver fibrosis. We also highlight the therapeutic potential of lncRNAs in chronic liver diseases.

## Introduction

Metabolic dysfunction-associated steatotic liver disease (MASLD) is the most recent term for metabolic syndrome-associated steatotic liver diseases, such as obesity and diabetes. The prevalence of MASLD has increased from 22% to 37% between 1991 and 2019 and is estimated to affect 30% of the global adult population,^1^ which parallels the increasing prevalence of obesity, diabetes, and relevant metabolic diseases. Metabolic dysfunction-associated steatohepatitis (MASH) is a more advanced stage of MASLD, which is defined by degenerative hepatocyte ballooning and lobular inflammation and is associated with a greater risk of liver fibrosis. MASH can further progress to liver fibrosis/cirrhosis, hepatocellular carcinoma (HCC), and liver failure, which are the leading causes of liver-related morbidity and mortality. Currently, there are no Food and Drug Administration or European Medicines Agency approved anti-fibrotic treatments available except the resmetirom for MASH, presenting an urgent need for the development of therapeutic strategies for MASH and liver fibrosis.^2^

### Risk factors for MASLD

Obesity and insulin resistance are two crucial metabolic risk factors contributing to the development of MASLD. These factors may be compounded by genetic abnormalities that dysregulate key lipid metabolic pathways and cause steatosis, for example, over-expression of genes involved in hepatic lipid synthesis (sterol regulatory element binding protein (*SREBP*), fatty acid synthase (*FAS*), stearoyl-coenzyme A (CoA) desaturase (*SCD*), etc.) and hepatic lipid uptake (low-density lipoprotein receptor, cluster of differentiation 36 *(CD36*)), or downregulation of genes required for hepatic lipid metabolism and transportation (apolipoprotein B), which is the toxic accumulation of lipids within hepatocytes.^3 4^

The majority of individuals with MASLD are obese. Notably, >95% of severely obese patients who undergo bariatric surgery have MASLD,^5^ demonstrating that obesity is a major epidemiological risk factor for MASLD. The increase in the incidence of obesity and MASLD is primarily attributed to factors such as unhealthy lifestyles and global economic development. Sedentary lifestyles and economic globalisation have influenced cultural shifts, leading to changes in dietary habits characterised by an increased consumption of refined sugars, processed foods, and calorie-dense additives.^6^ Lean MASLD, comprising 7%–20% of MASLD cases in various studies, was initially described in Asia but has since been observed in other populations globally. Despite having a body mass index (BMI) below the obesity threshold, individuals with lean MASLD exhibited similar patterns of fatty liver and metabolic abnormalities. Incidence studies of lean MASLD are limited but suggest annual incidence rates of 3%–5%, predominantly associated with fat mass expansion with increasing adiposity.^7^

Insulin resistance is another key contributor to MASLD and type 2 diabetes (T2D). There is a strong association between MASLD and T2D, with over 70% of patients with T2D also presenting with MASLD.^8^ Insulin resistance in hepatocytes results in lipid accumulation, which disturbs the hepatic metabolic system, for example, by upregulating hepatic de novo lipogenesis, altering the movement of fatty acids (FAs) from adipose tissue to the liver,^9^ and inhibiting mitochondrial FA β-oxidation, leading to injury of hepatocytes by lipotoxicity and the progression of MASLD to MASH and cirrhosis. Dysfunction of mitochondrial FA β-oxidation due to insulin resistance further induces mitochondrial oxidative stress,^10^ endoplasmic reticulum (ER) stress, and inflammatory responses, which, in turn, aggravates insulin resistance in hepatocytes by blunting the insulin signalling cascade.^9^ Additionally, glucotoxicity caused by chronic hyperglycaemia and impaired branched-chain amino acid metabolism in patients with T2D further promotes MASH progression via mammalian target of rapamycin (mTOR) signalling.^11^ Thus, T2D is a significant risk factor for MASLD development as it drives the progression of liver injury from simple steatosis to MASH and liver fibrosis.^8^

### Interaction between hepatocytes and non-parenchymal cells and pathophysiological signalling in the development of MASLD

The liver consists of four major cell types that play key roles in hepatic physiological and pathological functions and govern MASLD development. Hepatocytes account for approximately 60% of the total cells and 80% of the total liver volume. Hepatocytes are the predominant functional units of the liver in hepatic nutrient metabolism; biosynthesis of lipids, carbohydrates, and proteins; and detoxification owing to the large number of mitochondria, rough ER, Golgi apparatus, and free ribosomes. Hepatocytes are highly regenerative and contribute to the remarkable ability of the liver to recover from injury.^8^ Approximately 35% of the total liver cells and approximately 17% of the total liver mass comprise sinusoidal cells, including hepatic stellate cells (HSCs), liver sinusoidal endothelial cells (LSECs) and Kupffer cells. HSCs reside in the perisinusoidal space of Disse surrounded by hepatocytes and LSECs, and form the hepatic basement membrane by secreting type-IV collagen, laminin and proteoglycans.^12^ Of the liver cells, HSCs are predominantly associated with hepatic fibrosis. Loss of hepatocytes, inflammation and metabolic alterations induce trans differentiation of HSCs into proliferative, migratory and contractile myofibroblast-like cells (activated HSCs)^13^ that participate in hepatic fibrogenesis. Sinusoidal walls, which are physical barriers in the liver, consist of the second most abundant liver cell type, the LSECs.^14^ Kupffer cells are specialised hepatic macrophages that reside in the sinusoids, where they regulate hepatic and systemic immunological response to pathogens.^15^ Steatosis induces lipotoxicity and metabolic dysregulation, and activates pathophysiological signalling pathways in the liver, including oxidative and ER stress, metabolic inflammation, degenerative hepatocyte ballooning, apoptosis, and cell death, which is followed by the activation of fibrogenesis, leading to the initiation and progression of liver fibrosis/cirrhosis through complex processes involving nearby cells, such as HSCs, LSECs and Kupffer cells.

## Biogenesis And Characteristics Of Long Non-Coding Rnas

Only 1%–3% of the transcripts in the human genome are protein-coding transcripts that are translated into proteins.^16 17^ Recognition of non-coding RNAs as regulatory molecules began in early 2010 with the discovery of many small interfering RNAs (siRNAs), microRNAs (miRNAs) and small P-element induced wimpy testis (PIWI)-interacting RNAs (piRNAs) that regulate gene expression at the transcriptional, post-transcriptional, and translational levels in eukaryotes^18–20^ and bacteria.^21^ Recent studies have revealed that long non-coding RNAs (lncRNAs) are abundantly transcribed in the genome and exhibit a broad range of physiological and pathological functions via a variety of mechanisms. LncRNAs are defined as >200 nucleotide non-coding RNA transcripts which exclude miRNAs, siRNAs, piRNAs and the majority of infrastructural RNAs such as snoRNA, snRNA, rRNA and tRNA.^21^ More than 60000 lncRNAs have been identified within the human transcriptome, encompassing approximately 70% of the expressed genes. The significant number of lncRNAs in the human genome indicates their potentially crucial roles in human pathophysiological processes and, therefore, in human health and disease.

Most lncRNAs are transcribed by RNA polymerase II and are post-transcriptionally modified at the 5’ and 3’ ends. Compared with mRNAs, the majority of lncRNAs are retained within the nucleus, whereas only a fraction of lncRNAs are cytoplasmic.^22^ Based on their genomic localisation and nearby protein-coding genes, lncRNAs are commonly classified into five categories: (1) Intergenic (long intergenic non-coding RNAs), (2) Sense, (3) Antisense, (4) Bidirectional, and (5) Intronic.^23^

### Molecular functions of lncRNAs

Functionally, lncRNAs act as molecular guides, signalling molecules, decoys or scaffolds.^24^ The expression of lncRNAs in certain cell types and at different stages of development, in addition to their responses to external stimuli, endows lncRNAs with effective signalling potential. Functions of lncRNAs include binding to proteins to promote their degradation; recruiting regulatory molecules, eg, transcription factors, to specific gene promoters to control gene expression; and by associating with different domains of multiple molecules, lncRNAs can act as scaffolds in assembling a functional complex.^25^ Moreover, lncRNAs may also alter gene expression by histone modification, and a survey of 3000 human lncRNAs showed that approximately 24% of these human lncRNAs could physically bind to histone demethylating and trimethylating polycomb repressor 2 complexes (PRC2) via the PRC2 subunit enhancer of zeste homologue 2 (EZH2).^26^ The lncRNA phosphatase and actin regulator 1 (PHAC-TR)2-AS1 forms a complex with the RNA-binding protein vigillin and histone methyltransferase suppressor of variegation 3-9 homolog 1 (SUV39H1) to trigger histone H2 lysine 9 (H3K9) histone methylation, a process associated with gene downregulation.^27^ Furthermore, extensive research has focused on the sponging effects of lncRNAs on other RNAs, particularly on sponging miRNAs and modulating the actions of targeted miRNAs. The relevant lncRNAs are discussed below:

## Lncrnas target metabolic pathways associated with masld and liver fibrosis

In the liver, lncRNAs target the metabolic pathways involved in the development of MASLD and liver fibrosis. In hepatocytes, aberrant expression of lncRNAs modulates hepatic de novo lipid synthesis via mTOR, SREBBP1c/SREBP2, adenosine monophosphate (AMP)-activated protein kinase (AMPK) and sirtuin 6 (SIRT6), compromising FA β-oxidation via peroxisome proliferator activated receptor (PPAR)α; and perturbing hepatic glucose metabolism via forkhead box O1 (FOXO1), phosphoenolpyruvate carboxykinase, and glucose transporter (GLUT)2, which results in lipid accumulation and lipotoxicity in hepatocytes and proximate non-parenchymal cells. In HSCs, lncRNAs induce apoptosis and ubiquitination via p53, mouse double minute 2 homologue (MDM2), and phosphatase and tension homologue (PTEN) as well as activate fibrogenic and HSC proliferation via hedgehog (HH) signalling, wingless-related integration site (WNT)/β-catenin, small mothers against decapentaplegic homologue 2/3 and P21. In Kupffer cells, lncRNAs activate inflammatory pathways via nuclear factor-κB (NF-κB) and toll-like receptor (TLR)2/4. The molecular mechanisms of lncRNA-mediated liver injury or protection in the chronic liver metabolic diseases are summarised below.

### LncRNAs targeting metabolic pathways in hepatocytes contribute to MASLD and liver fibrosis

#### H19

H19, a lncRNA that spans 2.3kb and is situated at 11p15.5, is exclusively expressed from the maternal allele; H19 is detected in the nucleus and the cytoplasm but primarily functions in the cytoplasm.^28 29^ H19 expression is regulated by c-MYC, which binds to E-boxes near the imprinting control region, enabling histone acetylation and the initiation of H19 gene transcription in HCC cells.^30^ H19 upregulation is commonly observed in HCC associated with the Hepatitis B Virus (HBV), and it plays a significant role in genomic imprinting during growth and development.^31 32^

**Figure 1:**
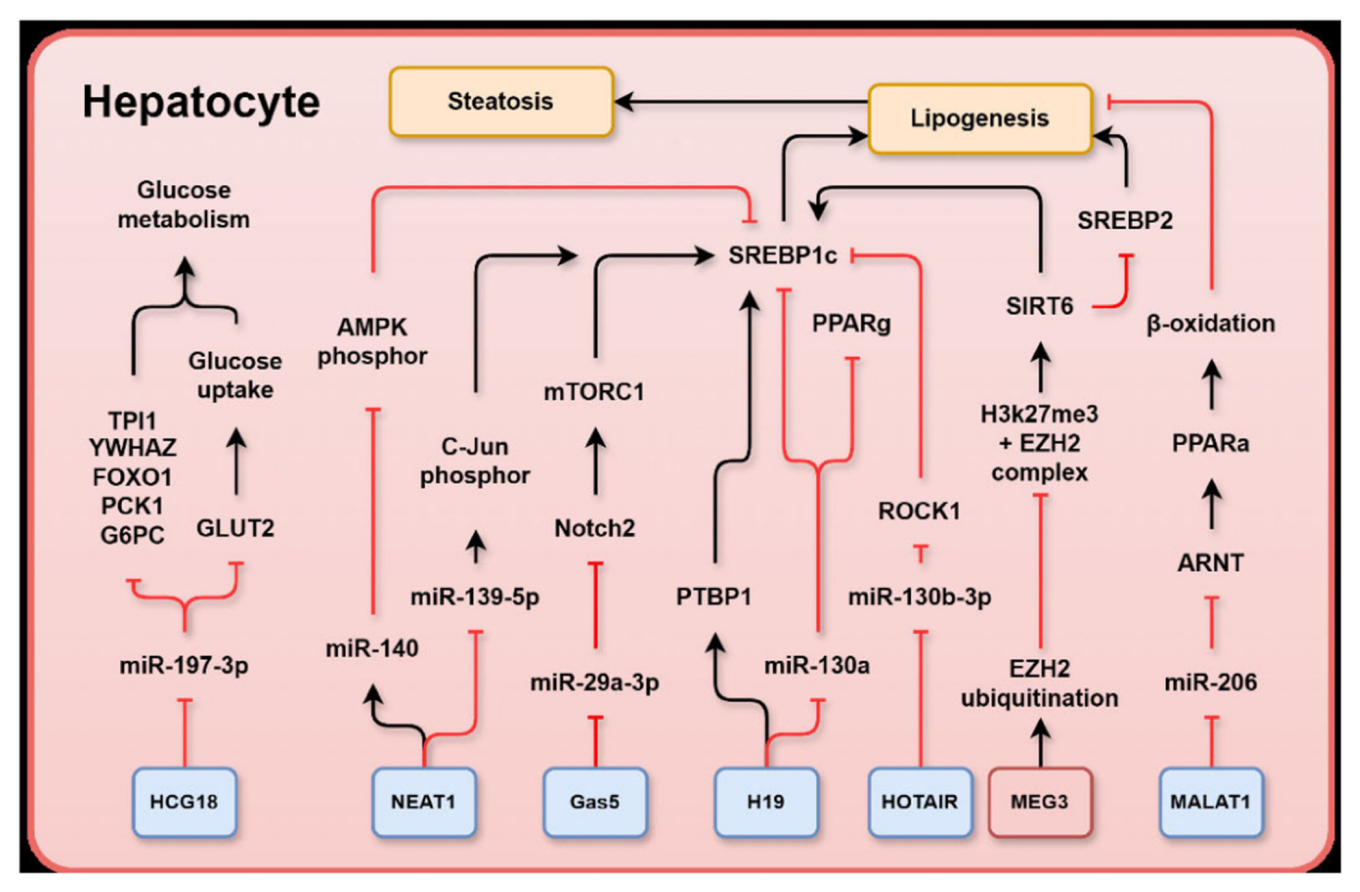
Schematic representation summarising MASLD-associated long non-coding RNAs (lncRNAs) and their relevant targets in hepatocytes: human leukocyte antigen (HLA) complex group 18 (HCG18), nuclear enriched abundant transcript 1 (NEAT1), growth arrest specific 5 (Gas5), H19 imprinted maternally expressed transcript (H19), homeobox transcript antisense RNA (HOTAIR), maternally expressed gene 3 (MEG3), metastasis-associated lung adenocarcinoma transcript 1 (MALAT1), triosephosphate isomerase 1 (TPI1), tyrosine 3-monooxygenase/tryptophan 5-monooxygenase activation protein zeta (YWHAZ), forkhead box O1 (FOXO1), phosphoenolpyruvate carboxykinase 1 (PCK1), glucose-6-phosphatase catalytic subunit (G6PC), adenosine monophosphate (AMP)-activated protein kinase (AMPK) catalytic subunit, Jun proto-oncogene (C-Jun), mammalian target of rapamycin complex 1 (mTORC1), neurogenic locus notch homolog protein 2 (Notch2), polypyrimidine tract-binding protein 1 (PTBP1), peroxisome proliferator activated receptor gamma (PPARγ), Rho-kinase 1 (ROCK1), sirtuin 6 (SIRT6), sterol regulatory element binding protein 2 (SREBP2), peroxisome proliferator activated receptor alpha (PPARα), aryl hydrocarbon receptor nuclear translocator (ARNT), polycomb repressive complex 2 subunit enhancer of zeste homologue 2 (EZH2), sterol regulatory element binding protein 1c (SREBP1c), glucose transporter 2 (GLUT2). LncRNAs in blue boxes are upregulated in MASLD whereas those in red boxes are downregulated. MASLD, metabolic dysfunction-associated steatotic liver disease.

In the context of MASLD, expression of H19 was increased in steatosis induced by oleic acid and by a high fat diet (HFD) in a mouse model during progression of MASLD.^33^ Overexpression of H19 in hepatocytes induced the expression of several genes involved in lipid synthesis, storage and breakdown, leading to lipid accumulation in the liver. In contrast, silencing of endogenous H19 reduced lipid accumulation in hepatocytes. The mechanism of H19 action depended on heterogeneous nuclear ribonucleoprotein A1 (hnRNPA1). Under fasting conditions, H19 induced cleavage of SREBP1 precursor and interacted with polypyrimidine tract-binding protein 1 to promote the translocation of SREBP1 to the nucleus (figure 1).^34^ SREBP1 is a master transcription factor that regulates de novo lipogenesis by targeting genes involved in FA synthesis including acetyl-CoA carboxylase (*ACC*) and *FAS*. Overexpression of SREBP promoted de novo lipogenesis and lipid accumulation in hepatocytes, leading to the progression of hepatic steatosis and disruption of metabolic pathways.^35^ Moreover, FAs exerted positive feedback regulation by inducing *hnRNPA1* and H19 expression. H19 was found to promote hepatic steatosis by increasing the expression of the lipogenic transcription factor MLX interacting protein like (ChREBP) and enhancing lipid accumulation in hepatocytes, which was effectively blocked by the phosphatidylinositol-4,5-bisphosphate 3-kinase (PI3K)/mTOR inhibitor PF-04691502. Consistently, overexpression of H19 in hepatocytes led to the upregulation of key components of the mTOR complex 1 (mTORC1) signalling pathway, which was inhibited when endogenous H19 was silenced,^33^ indicating that mTORC1 signalling is involved in H19-induced lipid synthesis.

Alternative mechanisms of H19 in MASLD development involved the mediation of PPARγ/miR-130a axis (figure 1). In this regulation, PPARγ, a nuclear receptor highly expressed in adipose tissue was upregulated and activated in patients with MASLD in association with increased H19 *and* downregulation of miR-130a. It was shown that miR-130a and H19 were able to directly bind to one another and miR-130a was an inhibitor of PPARγ expression. Additionally, miR-130a expression was associated with downregulation of the lipogenesis genes *Fasn, Acc, Srebp1* and *Scd1*; therefore, the inhibitory action of H19 on miR-130a promoted steatosis. Given the interaction between lncRNA H19, hnRNPA1 protein, PPARγ and miR-130a, it can be inferred that H19 is a crucial lncRNA in the development of fatty liver and steatosis.^36^

Additionally, H19 functioned as a competitive endogenous RNA that binds to zinc finger E-box homeobox 1 (ZEB1), leading to increased epithelial cell adhesion molecule (EpCAM) expression and promotion of cholestatic hepatic fibrosis. It has been reported that H19 expression increased B cell lymphoma-2-induced cholestatic liver injury, and that injury could be mitigated by reducing H19 expression. It was also shown that ZEB1 inhibited EpCAM promoter activity and gene transcription. Upregulation of H19 RNA curbed ZEB1 expression and its binding to the EpCAM promoter, led to the de-repression of EpCAM by ZEB1 inhibition and enhanced EpCAM transcription.^37^

### Growth arrest specific 5

Growth arrest specific 5 (Gas5) is an lncRNA enriched in the nucleus and cytoplasm and is encoded on chromosome 1q25 in a gene of 630 nucleotides with 12 exons; it is a ribo-suppressive lncRNA that acts as a decoy for the glucocorticoid receptor by imitating the glucocorticoid response element present in genomic DNA. Expression of Gas5 was elevated in humans and mice with MASLD. Gas5 was found to sponge miR-29a-3p and suppressed its negative regulation on the neurogenic locus notch homologue protein 2 (*Notch2*), a known promoter of hepatic steatosis, leading to the upregulation of Notch2. Inhibition of Notch signalling was found to ameliorate hepatic steatosis by inhibiting mTORC1, a protein previously shown to be a protective regulator of lipid homoeostasis in an S6K-dependent and protein kinase B (AKT)-independent manner,^38^ and augmenting SREBP1c-mediated lipogenesis^39^ (figure 1). Gas5 knockdown attenuated HFD-induced hepatic steatosis, reduced plasma triglyceride and cholesterol levels, and inhibited alanine aminotransferase and aspartate aminotransferase activities, all of which contributed to the reduced severity score of MASLD.^40^ The expression of Gas5 was significantly higher in the tissues and plasmas of patients with advanced fibrosis than in those without fibrosis. However, patients with cirrhosis were an exception with plasma Gas5 lower in patients with cirrhosis than in patients with advanced fibrosis,^41^ which could be the consequence of functional decompensation at the cirrhosis stage.

**Figure 2:**
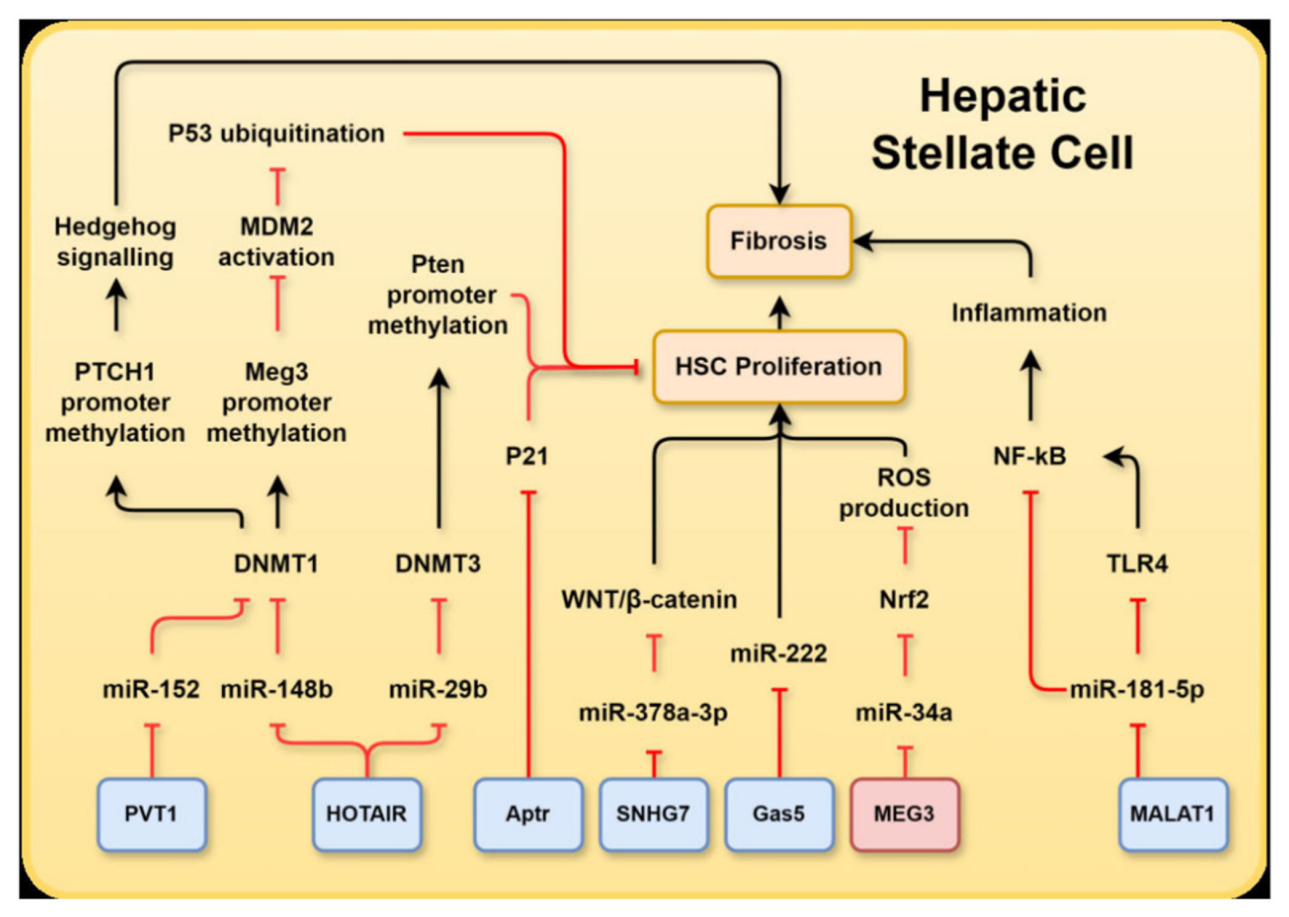
A schematic representation summarising MASLD-associated long non-coding RNAs (lncRNAs) and their associated relevant targets in hepatic stellate cells (HSCs): plasmacytoma variant translocation 1 (PVT1), homeobox transcript antisense RNA (HOTAIR), alu-mediated P21 transcriptional regulator (APTR), small nucleolar RNA host gene 7 (SNHG7), growth arrest specific 5 (Gas5), maternally expressed gene 3 (MEG3), metastasis-associated lung adenocarcinoma transcript 1 (MALAT1), DNA (cytosine-5-)-methyltransferase (DNMT), patched 1 gene (PTCH1), mouse double minute 2 homologue (MDM2) proto-oncogene, phosphatase and tensin homolog (Pten), nuclear factor erythroid 2-like basic leucine zipper transcription factor 2 (Nrf2), reactive oxygen species (ROS), nuclear factor-κB (NF-κB), toll-like receptor 4 (TLR4). LncRNAs in blue boxes are upregulated in MASLD whereas those in red boxes are downregulated. MASLD, metabolic dysfunction-associated steatotic liver disease.

miR-222 is one of the most studied miRNAs involved in the progression of MASLD and hepatic fibrosis. As MASLD progresses to fibrosis, miR-222 expression increased in activated HSCs. Gas5 is a competing endogenous RNA for miR-222; miR-222 was able to inhibit the expression of Gas5 and Gas5 was able to suppress miR-222 expression (figure 2). Through this mechanism, Gas5 overexpression inhibited the activation and proliferation of HSCs in a reversible manner.^42^ This is consistent with recent research showing Gas5 overexpression in human HSC cell line (LX-2) cells reduced expression of α-smooth muscle actin (αSMA) and collagen type 1 alpha 1 (COL1A1) in addition to reducing cell proliferation by inducing G0/G1 phase arrest. It was proposed that this effect was caused by Gas5 overexpression, which reduced the levels of insulin-like growth factor 1 (IGF1), phosphorylated-PI3K and phosphorylated p-AKT.^43^

### Homeobox transcript antisense RNA

Homeobox transcript antisense RNA (HOTAIR), an lncRNA of 2158 nucleotides in length, is transcribed in proximity to the *HOXC* genes on chromosome 12q13.13. It functions as a polycomb group protein that suppresses the transcription of numerous genes that play a role in stem cell pluripotency and various developmental differentiation pathways.^44^

Research conducted by Bian *et al* revealed a significant increase in the expression of HOTAIR in human fibrotic livers, and mouse models of hepatic fibrosis induced by carbon tetrachloride (CCl_4_) or transforming growth factor (TGF)β−1 induced activation of HSCs. HOTAIR directly bound to miR-148b, thereby regulating the DNA (cytosine-5-)-methyltransferase 1 (DNMT1)/maternally expressed gene 3 (MEG3)/p53 pathway in HSCs^45^ (figure 2). DNMT1 was a positive regulator of DNA methylation and could induce hypermethylation of the miR-148a promoter. In contrast, miR-148a targeted DNMT1 and reduced its abundance of DNMT1. Thus, a negative feedback loop exists between miR-148a and DMNT1. DNMT1 downregulation reduced *MEG3* expression by inhibiting *MEG3* promoter methylation. MEG3 suppressed activation of MDM2 and upregulated P53. Reduced expression of MEG3 resulted in rapid ubiquitination of P53 via MDM2.^46^ This ultimately promoted HSC survival and enhanced hepatic fibrosis. HOTAIR was also involved in gene silencing through its interaction with the chromatin remodelling factor PRC2 and contributed to liver cirrhosis^45 47^ (figure 2).

PTEN is a phosphatase that functions as a tumour suppressor and negatively regulated insulin signalling. Expression of PTEN was downregulated in liver exposed to free fatty acids (FFAs) via mediation of the NF-κB/mTOR pathway. Studies have demonstrated that miR-29b played a role in preventing liver fibrosis by reducing the activation of HSCs and promoting apoptosis by targeting the PI3K/AKT pathway.^48^ Yu *et al* reported that HOTAIR expression increased in HSCs in vivo and in vitro during hepatic fibrosis. HOTAIR was shown to be a target of miR-29b, and these RNAs mutually inhibited each other. Sponging of miR-29b inhibited the methylation of the PTEN promoter by miR-29b target DNMT3b (figure 2). Consequently, PTEN expression was reduced by HOTAIR overexpression, and the reduced expression of this tumour suppressor promoted proliferation of HSCs.^49^ These findings demonstrated that the HOTAIR/miR-29b/PTEN functional network is involved in hepatic fibrosis.

Rho-kinase 1 (ROCK1) is a known driver of HFD/obesity-induced hepatic steatosis via stimulation of de novo lipogenesis. In the livers of human subjects with MASLD, ROCK1 expression and activity were significantly increased by approximately 2.2-fold compared with healthy controls. Notably, ROCK1 deficiency protected against obesity-induced metabolic disorders and prevented hepatic steatosis in mice, which was associated with the downregulation of lipogenic genes, including *FAS, SCD1, SREBP1C* and *ELOVL2*.^50^ In this regulation, HOTAIR inhibited the function of miR-130b-3p,^51^ thereby preventing the inhibition of ROCK1 by miR-130b-3p (figure 1). Consequently, overexpression of HOTAIR induced hepatic steatosis via miR-130b-3p-mediated inhibition of ROCK1.^51^

HOTAIR was also found to directly interact with serinerich and arginine-rich splicing factor 1 (SRSF1) in the liver, which induced hepatic inflammation and fibrosis via global disruption of transcription and inhibition of protein translation.^52^ The interaction between HOTAIR and SRSF1 further increased the stability of the helixloop-helix leucine zipper transcription factor of the Myc/Max/Mad superfamily and ChREBP, accelerating inflammation and progression of MASLD.^53^

### Maternally expressed gene 3

MEG3 is a nuclear retained lncRNA encoded by chromosome 14q32.3 with a length of 35 kb and consisting of 10 exons; loss of MEG3 has been linked to various human cancers, and several studies have uncovered the downregulation of MEG3 in MASLD mouse models. Overexpression of MEG3 in primary hepatocytes protected cells from steatosis by reducing FFA accumulation via the redirection of EZH2 to ubiquitination-mediated proteolysis, consequently reducing the binding of EZH2 and H3K27me3 to the promoter of sirtuin 6 (*SIRT6*), a gene previously shown to be protective against MASLD^54 55^ (figure 1). MEG3 further reduced miR-34a levels via sponging, which de-repressed the inhibition of miR-34a on nuclear factor erythroid 2 related factors and promoted the removal of reactive oxygen species and reduced cellular apoptosis^56^ (figure 2). Therefore, MEG3 exerted a protective effect against hepatic ischaemia reperfusion.

Reduced MEG3 expression was found in the livers of CCl_4_-treated mice and was negatively correlated with fibrosis progression.^57^ MEG3 was shown to co-localise with αSMA in fibrotic mouse livers, indicating HSCs as a source of this lncRNA.^57^ Functional assays in human HSCs (LX-2 cells) further uncovered that TGFβ1 mediated downregulation of MEG3 in a time-dependent and dose-dependent manner, whereas MEG3 overexpression inhibited TGFβ1-induced cell proliferation and promoted apoptosis via p53 and cytochrome-C pathways. Hepatic MEG3 levels were reduced in human livers with fibrosis and cirrhosis.^58^ These data suggested that loss of MEG3 played a crucial role in the development of hepatic steatosis in the early stages of MASLD via mediation of SIRT6. The inverse relationship between MEG3 and TGFβ1 further suggested the protective effect of MEG3 could extend to hepatic fibrosis in which MEG3 suppressed αSMA expression by indirectly antagonising TGFβ1.

### Nuclear enriched abundant transcript 1

Nuclear enriched abundant transcript 1 (NEAT1) is a nuclear retained lncRNA expressed ubiquitously throughout the body with two transcripts: NEAT1_1 and NEAT1_2. The longer variant, NEAT1_2 (22 741 bp), is essential for assembly of nuclear paraspeckles, the 0.2–1 µm ribonucleoprotein bodies located within the interchromatin space of mammalian nuclei. Paraspeckles regulated gene expression by retaining RNA in the nucleus, sequestering nuclear proteins and altering epigenetic histone markers. The other transcript, NEAT1_1 (3735 bp), was also a component of paraspeckles, but was dispensable for their formation. In unstressed cells, NEAT1 was localised in the nucleus. However, in response to inflammation, NEAT1 was released from the nucleus and translocates to the cytoplasm.^59^ NEAT1 was upregulated in both in vivo and in vitro models of MASLD.^60^ NEAT1 expression was positively correlated with the activation of Jun protooncogene (c-Jun) and SREBP1c but inversely associated with miR-139–5 p expression in MASLD^60^ (figure 1). miR-139–5 p has been proposed to inhibit c-Jun activation and the expression of genes involved in de novo lipid synthesis, including *SREBP1c, FASN*, and *ACC*.^60^ NEAT1 knockdown prevented lipid accumulation in hepato-cytes and alleviated MASLD by inhibiting mTOR/S6K1.^61^ Additionally, NEAT1 was further found to interact with oestrogen receptor α and promoted steatosis in human hepatoblastoma cell line (HepG2) cells.^62^

Additionally, NEAT1 expression was elevated in liver tissues and primary HSCs derived from fibrotic mouse livers induced by CCl_4_ treatment. Knockdown of NEAT1 in primary HSCs inhibited cell proliferation by 54% and suppressed expression of fibrogenic molecules, αSMA and COL1A1, resulting in improvement of liver fibrosis. Conversely, NEAT1 overexpression stimulated HSC activation and increased αSMA and COL1A1 levels, indicating the involvement of NEAT1 in HSC activation and liver fibrosis. Another study reported that NEAT1 overexpression correlated with reduced miR-122 abundance which regulates NEAT1 and HSC activation through Kruppel-like factor 6 (KLF6). Regulation of the NEAT1-miR-122-KLF6 axis in chronic liver disease has been observed in the liver tissues of patients with MASH and advanced fibrosis, in which NEAT1 and KLF6 were increased, whereas miR-122 was decreased. These observations further supported the involvement of NEAT1 in liver fibrogenesis in humans and mice.

NEAT1 has also been found to couple with miR-140 and synergistically exacerbated MASLD. Both NEAT1 and miR-140 were upregulated in MASLD and suppression of either miR-140 or NEAT1 alleviated MASLD. Silencing NEAT1 downregulated miR-140 and vice versa. It has been shown that knockdown of miR-140 or NEAT1 increased phosphorylation of AMP-activated protein kinase (AMPK), which inhibited expression of genes involved in de novo lipid synthesis, eg, *SREBP1, FAS* and *ACC*^63^ (figure 1). Chen *et al* reported that NEAT1 downregulation promoted FFA uptake in hepatocytes and decreased the expression of the key FA transmembrane protein CD36. Inhibition of NEAT1 by short hairpin RNA reduced phosphorylation of AMPK and carnitine palmitoyltransferase (CPT1) but enhanced the expression of *SREBP1c, FAS* and *ACC* in FFA-treated human hepatocytes, HepG2. Increased NEAT1 sponges miR-146a-5p, thus releasing its inhibition on ROCK1, a kinase that promotes obesity-associated MASLD.^64^

The transcription factor zinc finger protein 143 (ZNF143) was upregulated by FFA and was overexpressed in MASLD. ZNF143 is a transcription factor for NEAT1, and the upregulation of NEAT1 by ZNF143 exacerbated steatosis. In this pathway, NEAT1 targeted the RNA-binding protein staphylococcal nuclease domaincontaining 1 (SND1) and promoted ROCK2 in HepG2 and human hepatoma cell line (Huh-7) cells. ROCK2 is involved in a broad range of biological pathways, including lipid metabolism, inflammation and mitophagy inhibition. ZNF143/NEAT1/SND1-mediated ROCK2 signalling upregulation was associated with the downregulation of mitophagy proteins PTEN-induced kinase 1 (PINK1) and Parkin, suggesting that NEAT1 overexpression may contribute to steatosis by impairing mitochondrial repair mechanisms.^65^

NEAT1 expression positively correlated with glutamate ionotropic receptor AMPA type subunit 3 (GRIA3) expression and inversely with *miR-212–5* p expression. FFA accumulation promoted NEAT1 and GRIA3 expression whilst suppressing miR-212–5 p expression in hepatocytes. In this mechanism, miR-212–5 p was a direct target of NEAT1 while miR-212–5 p targeted GRIA3. FFA-induced H3K27 acetylation at the NEAT1 promoter, which upregulated NEAT1 in this regulatory network.^66^ Furthermore, NEAT1 played a regulatory role in hepatic fibrosis by promoting the expression of GLI3, a downstream gene of the HH fibrosis pathway, whose expression was inversely related to miR-506 expression in MASLD. NEAT1 knockdown inhibited GLI3 expression and promoted miR-506 expression, whereas miR-506 overexpression inhibited NEAT1 and GLI3 expression.^67^

### LncRNA metastasis-associated lung adenocarcinoma transcript 1

Metastasis-associated lung adenocarcinoma transcript 1 (MALAT1) is an lncRNA with approximately 8.7 Kb length. MALAT1 is a highly conserved vertebrate lncRNA. Processing of the MALAT1 nascent transcript yields a long nuclear-retained non-coding RNA (ncRNA) and a transfer RNA (tRNA)-like tail from the 3’ end which translocates to the cytoplasm. MALAT1 expression was induced and positively associated with the pathological progression of MASLD and hepatic fibrosis. Serum MALAT1 level was also positively correlated with inflammatory cytokines, tumour necrosis factor (TNF)α, interleukin (IL)-6 and high sensitivity C-reactive protein (hs-CRP).^68^ MALAT1 knockdown ameliorated FFA accumulation in hepatocytes which was in part mediated by MALAT1 sponging of miR-206 and the subsequent release of its inhibition of the target gene, the aryl hydrocarbon receptor nuclear translocator (*ARNT*). Increased ARNT promoted hepatic lipid accumulation by interacting with PPARα promoter and inhibiting FA β-oxidation (figure 1). Elevated MALAT1 further interacted with the FA transporter CD36^69^ and was involved in hepatic lipid droplet metabolism. CD36 is a membrane-localised lipid sensor that catalyses the initial step of triglyceride hydrolysis in hepatic lipid droplets. Knockdown of CD36 has been shown to upregulate PPARα expression and prevent hepatic lipid accumulation. Increased MALAT1 enhanced expression of CD36 and disruption of lipid metabolism via inhibition of PPARα whereas knockdown of MALAT1 upregulated PPARα expression and suppressed CD36 expression.^69^

Further study revealed that knockdown of MALAT1 was associated with reduced expression of fibrogenic genes *αSMA* and *COL1A1* in primary mouse HSCs, which prevented transformation of HSCs into myofibroblast-like morphology, a characteristic of HSC activation. In a mouse model of CCl_4_-induced liver fibrosis, MALAT1 expression increased approximately 5.9-fold in HSCs and 2.7-fold in hepatocytes compared with that in controls. Knockdown of MALAT1 reduced collagen accumulation by 54% in mouse liver, which was mediated by the sponging interaction between MALAT1 and miR-181a-5p (figure 2). Specifically, the authors showed that expression of inflammatory molecules, NF-κB and TLR4, was negatively associated with miR-181a-5p but positively correlated with MALAT1 level in cells treated with lipopolysaccharide (LPS) or TGFβ1. MiR-181a-5p targeted the 3’-UTR of TLR4 and inhibited TLR4-mediated inflammatory signalling (figure 2). MiR-181a-5p was reported to further target the high mobility group box 1, a known activator of NF-κB signalling, or directly target the miRNA binding site within 3’-UTR of NF-κB, and negatively regulated NF-κB signalling.^70^ Knockdown of MALAT1 or overexpression of miR-181–5 p was able to ameliorate TGFβ1-induced viability, proliferation, migration, adhesion and collagen production in LX-2 HSCs treated with LPS.^68^ Moreover, MALAT1 was further found to sequester miR-101b and reduce its inhibitory effect on Ras-related C3 botulinum substrate 1 (Rac1) within HSCs,^71^ leading to increased expression of Rac1 and activation of HSCs and fibrogenic TGFβ1 signalling.^72^

### HCG18

Human leukocyte antigen (HLA) complex group 18 (HCG18) is located on the human chromosome 6p22.1, and four transcripts have been reported (NR _0024053.2, NR_102326.1, NR_024052.2 and NR_102327.1). Expression levels of HCG18 were higher in patients with MASLD compared with that in controls, and in individuals with a Homeostasis Model Assessment of Insulin Resistance (HOMA-IR) score ≥2.5 compared with those with a score <2.5. In patients with MASLD, HCG18 expression correlated with BMI, HOMA-IR, alanine aminotransferase (ALT), fasting blood glucose, total cholesterol and triglycerides. Additionally, HCG18 exerted protective effects on blood glucose levels and fat deposition but not on body weight. MiR-197–3 p was identified as a direct target of HCG18, and a negative correlation was observed between miR-197–3 p and HCG18 expression. Moreover, the effect of HCG18 on insulin resistance and lipid accumulation was counteracted by miR-197–3 p expression.^73^ Hu *et al* showed that miR-197–3 p negatively regulated cellular glucose metabolism and insulin sensitivity by targeting TPI1 in plasma and HepG2 cells, in addition to its regulation of YWHAZ (tyrosine 3-monooxygenase/tryptophan 5-monooxygenase activation protein zeta) and FOXO1 (figure 1), the downstream molecules of the PI3K-AKT pathway.^74^ Additionally, the same group observed that miR-197–3 p indirectly inhibited the transcription of GLUT2 mRNA and decreased cellular glucose uptake (figure 1).

### LncRNAs targeting inflammatory and fibrogenic pathways in HSCs contribute to MASLD and liver fibrosis

#### Plasmacytoma variant translocation 1

Plasmacytoma variant translocation 1 (PVT1) is a 1716 nucleotide lncRNA presented in the cytoplasm and nucleus. PVT1 expression was positively associated with genes involved in fibrogenesis, including *PAI-1, TGFβ1*, fibronectin 1 and *COL4A1*, and was inversely associated with bone morphogenic protein 7 (BMP7) level. The inhibition of PVT1 offered a protective approach against fibrosis. Silencing PVT1 reduced extracellular matrix accumulation and TGFβ1 expression in a model of diabetic nephropathy. Zheng *et al* showed a 14.1-fold increase in PVT1 expression in the liver of a CCl_4_-induced liver fibrosis mouse model, whereas PVT1 knockdown reduced hepatic collagen expression. Research from both in vitro and in vivo studies further supported that reduced lncRNA PVT1 expression helped prevent fibrosis. Knock-down of PVT1 in primary HSCs inhibited cell proliferation, reduced expression of *αSMA* and *COL1A1*, and altered expression of epithelial-mesenchymal transition markers, E-cadherin, desmin and vimentin.^75^

Interactions between PVT1 and miR-152 contributed to regulation of HSC activation. miR-152 reduced the methylation of patched 1 gene (PTCH1) by targeting and negatively regulating DNMT1. Reduced methylation of PTCH1 inhibited the activation of HH signalling and epithelial-to-mesenchymal transition in HSCs (figure 2). PVT1 is a sponge for miR-152; therefore, upregulation of PVT1 activates HH signalling by impeding the inhibitory effect of miR-152 on HH signalling activation (figure 2).^75^ Furthermore, PVT1 was a sponge for miR-20a-5p, a miRNA that was typically reduced in the plasma of patients and mice with MASLD. miR-20a-5p was an inhibitor of CD36, and the inhibition of CD36 by miR-20a-5p has been shown to reduce lipid accumulation in HepG2 cells. Elevated PVT1 expression in MASLD enhanced lipid accumulation by enhancing CD36 expression via miR-152 downregulation.^76 77^

#### Alu-mediated P21 transcriptional regulator

Alu-mediated P21 transcriptional regulator (APTR) is a 2303-nucleotide lncRNA present in both the cytoplasm and nucleus, and was found to positively regulate cell cycle progression and cell proliferation via suppression of cyclin-dependent kinase inhibitor 1A (P21). This occured due to the direct interaction between APTR and PRC2 and recruitment of PRC2 to the *P21* promoter, which resulted in methylation of histone H2 at lysine 27, downregulating the transcriptional activity of this gene^78^ (figure 2).

Elevated APTR expression was observed in two mouse models with liver fibrosis induced by CCl_4_ and bile duct ligation, as well as in humans with liver fibrosis of an unspecified aetiology. Knockdown of APTR using adenovirus in CCl_4_-treated mice resulted in reduced levels of αSMA and COL1A1, and mitigated liver fibrosis. Similarly, The TGFβ1-induced upregulation of αSMA, and the overexpression of *αSMA* and *COL1A1* was attenuated at mRNA and protein levels by APTR knockdown in primary HSCs. Patients with liver cirrhosis with diseases of unspecified aetiology showed serum levels of APTR approximately fourfold higher than those in histologically healthy individuals and twice as high in patients with decompensated cirrhosis as those in compensated cases, indicating its potential as a biomarker for liver fibrosis severity. Given the weak primary sequence conservation of lncRNAs compared with protein-coding genes, the consistent APTR expression patterns observed in mice and humans across different aetiologies of liver fibrosis indicate the potentially crucial role of APTR in liver fibrosis. Future studies, including the elucidation of APTR’s role in HSC activation and proliferation, merit further investigation. Analysis of serum APTR in a larger cohort covering a broader spectrum of fibrosis severity (mild to severe) will further contribute to our understanding of the involvement of this lncRNA in MASLD-associated fibrogenesis.^79^

#### Small nuclear RNA host gene-7

Small nuclear RNA host gene-7 (SNHG7) is encoded on chromosome 9q34.3 and is 2176 bps in length. It is predominantly localised to the cytoplasm of human hepatocytes (HepG2).^80^ The expression of SNHG7 was significantly increased in human fibrotic liver tissues compared with that in healthy controls. SNHG7 over-expression led to increased expression of WNT/β-catenin pathway proteins T cell factor and P-β-catenin and glycogen synthase kinase-3β. SNHG7 acted as a sponge for miR-378a-3p, which reduced the inhibitory effect of miR-378a-3p on a WNT/β-catenin component (figure 2), dishevelled segment polarity protein 2 (DVL2). Reduced SNHG7 expression inhibited the activation of HSCs and fibrosis in mice. Loss of SNHG7 contributes to reduced HSC activation, partially via miR-378a-3p and DVL2.^81^

### LncRNAs targeting inflammatory signalling in Kupffer cells contribute to MASLD and liver fibrosis

#### LncRNA-FTX

**Figure 3:**
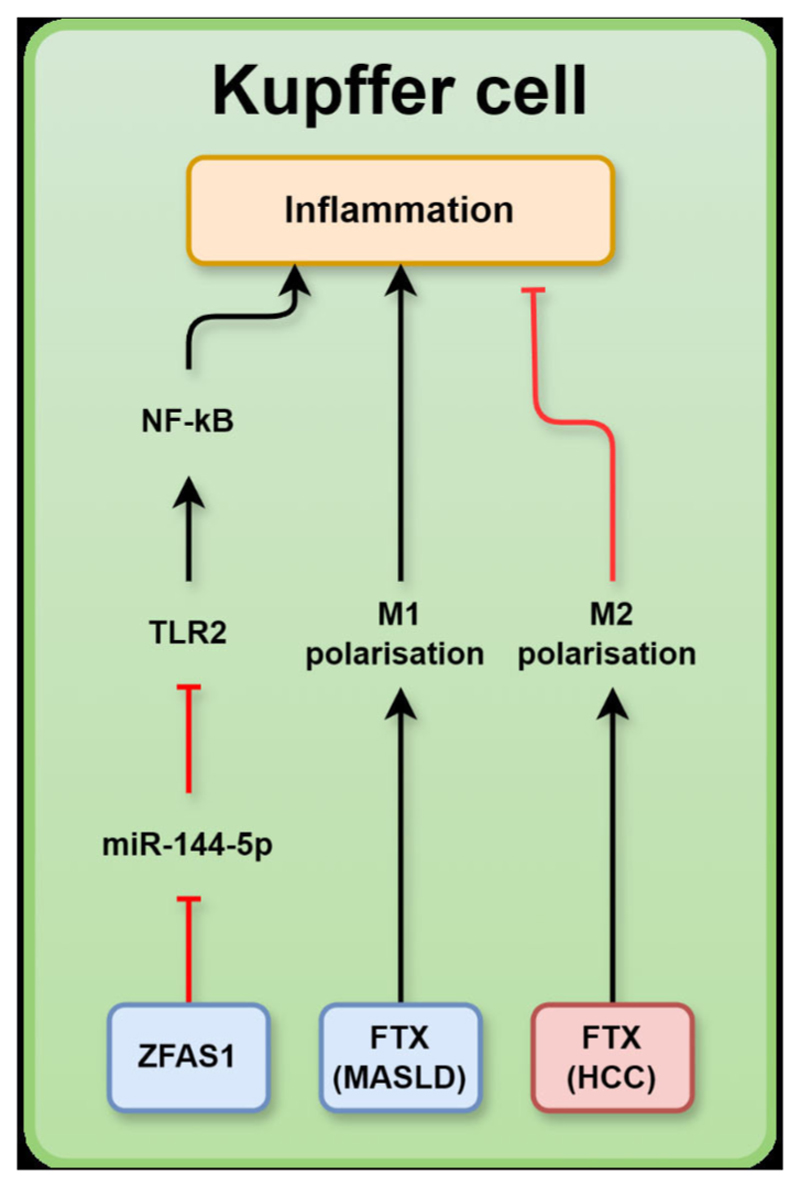
A schematic representation summarising MASLD-associated long non-coding RNA (lncRNAs) and their relevant targets in Kupffer cells: ZNFX1 antisense RNA 1 (ZFAS1), five prime to XIST (FTX), nuclear factor-κB (NF-κB), toll-like receptor 2 (TLR2) in metabolic dysfunction-associated steatotic liver disease (MASLD) or hepatocellular carcinoma (HCC). LncRNAs in blue boxes are upregulated in MASLD whereas those in red boxes are downregulated. MASLD, metabolic dysfunction-associated steatotic liver disease; XIST, X interactive specific transcript.

LncRNA-5 primed to X interactive specific transcript (*XIST)* (FTX) is 2300 nucleotides in length and is encoded upstream of *XIST*. In mice and humans, FTX is evolutionarily conserved and encoded on chromosome Xq13.2.^82^ This lncRNA has been observed in the nucleus and cytoplasm, and its expression was upregulated in the liver tissue of HFD-induced MASLD mice; however, it was lower in patients with HCC than in those with MASLD.^83^ FTX expression has been associated with the progression of MASLD to HCC in patients with MASLD, with research showing that the upregulation of FTX promoted HCC growth by promoting M1 polarisation of Kupffer cells. Notably, downregulation of FTX promoted the M2 polarisation of Kupffer cells, causing a decrease in the ratio of M1:M2 Kupffer cells in MASLD-HCC liver tissue^84^ (figure 3). M1 polarised Kupffer cells are ‘classically activated’ and produce proinflammatory cytokines, whereas M2 Kupffer cells are ‘alternatively activated’ and produce anti-inflammatory cytokines. The pro-inflammatory activity of M1 polarised Kupffer cells attracts immune cells to sites of tumour growth to kill the cancerous cells. In contrast, the secretion of anti-inflammatory cytokines by M2 polarised Kupffer cells in areas of tumour growth can create an anti-immunogenic tumour microenvironment that promotes tumour growth by preventing immune cells from killing cancerous cells.^85^

In hepatic steatohepatitis, upregulation of NF-κB drove Platr4 expression, which served as a negative feedback regulator to suppress the nucleotide-binding oligomerisation domain-like receptor family pyrin domain containing 3 (NLRP3) inflammasome via inhibition of NF-κB-dependent transcription of NLRP3 and caspase recruitment domain-containing protein 5 (ASC) in macrophages. This negative regulation of NF-κB occurred via interaction between Platr4 and the retinoid x receptor (RXR)α protein, which interfered with the interaction between NF-κB P65/RXRα complex and κB consensus sites in the nucleus.

#### LncRNA ZFAS1

Elevated expression of lncRNA ZNFX1 antisense RNA 1 (ZFAS1) was observed in patients with MASLD. ZFAS1 has five transcript variants, with lengths ranging from 504 to −1008 bp. In an HFD-induced MASLD mouse model, suppression of ZFAS1 reversed HFD-induced serum concentrations of anti-inflammatory cytokine IL-10 but reduced proinflammatory cytokine TNFα. Further studies revealed that ZFAS1 inhibits miR-144–5 p via sponging (figure 3). Indeed, miR-144–5 p was down-regulated in the livers of human patients with MASLD.^86^ The downregulation of miR-144 was shown to enhance expression of TLR2 and upregulation of inflammatory signalling proteins, including TNFα, interferon-γ and NF-κB in hepatic Kupffer cells^87^ (figure 3).

## Therapeutic and diagnostic potential of lncrnas

Currently, no approved medicines are available for the treatment of MASLD or liver fibrosis except the resmetirom for MASH. Translational research of the therapeutic potential of lncRNAs in clinical treatment has been challenging because of species specificity and reduced conservation of lncRNA sequences among mammals. Many lncRNA sequences are poorly conserved between humans and rodents making murine models unsuitable for some, but not all, lncRNA research. Species that are more closely related to humans (eg, primate and ape models) may be more suitable for preclinical studies. Therefore, it appears that the lncRNAs available for clinical trials are broadly conserved between humans and animals. Nevertheless, several lncRNAs have been successfully developed for cancer therapy and have entered clinical trials. For instance, a clinical trial completed in April 2022 by researchers at Mansoura University (Egypt) investigated the association between lncRNA H19 and IGF1 receptor gene expressions in patients with HCC and T2D to uncover the underlying pathophysiological link between HCC and T2D, which may become a therapeutic target for both diseases (NCT04767750). However, these results have not yet been published. Additionally, a clinical trial (NCT05088811) sponsored by Alexandria University (Egypt) investigated the role of lncRNAs WD repeat-containing antisense to TP53 (WEAP53) and urothelial carcinoma-associated-1 as potential biomarkers for the diagnosis of HCC. Another ongoing clinical trial was conducted by researchers at Tongji Medical College in China, which investigated alterations in the gut microbiota composition of circulating biomarkers (ie, lncRNAs) for drug-induced liver injury (NCT05465642). Circulating lncRNAs targeting miR-146 were the topic of interest in a clinical trial which ended in 2022 and investigated the effect of lncRNAs on T2D peripheral neuropathy (NCT04638556); however, these results remain unpublished.

Clinical diagnostic techniques require simple and preferably non-invasive techniques, low cost, and accurate and reproducible results. Currently, the diagnosis of MASLD requires a simple blood test called the liver function test. Collection of liver tissue to analyse changes in lncRNA expression is more complex and invasive. However, there are lncRNAs that are secreted into circulation at varying concentrations depending on disease status. For example, NEAT1 is upregulated in the serum and peripheral blood mononucleocytes of patients with MASLD. Thus, it is optimal to expect that circulating lncRNAs may serve as diagnostic and therapeutic biomarkers in metabolic disease treatment and prognosis.^88^

## Conclusion And Future Perspectives

The relatively recent discovery of lncRNAs has revealed a complex network of non-coding RNA interactions that underlies the development of a number of human diseases. Currently, only three clinical trials of lncRNAs in HCC therapy are listed in the US National Library of Medicine database for clinical trials. Technological and computational advances will enable the novel functional annotation of lncRNAs and offer revolutionary treatments for human diseases, including MASLD and liver fibrosis.
