# Supplementary material for: Mechanistic role of long non-coding RNAs in the pathogenesis of metabolic dysfunction-associated steatotic liver disease and fibrosis
Source: eGastroenterology. 2024 Nov 18;2(4):e100115. doi: 10.1136/egastro-2024-100115 (PMC11729351; doi:10.1136/egastro-2024-100115)
Supplement: Supplementary file 1 [file egastro-2024-100115.reviewer_comments.pdf]

PEER REVIEW HISTORY

eGastroenterology publishes all reviews undertaken for accepted manuscripts. Reviewers are asked to complete a checklist review form and are provided with free text boxes to elaborate on their assessment. These free text comments are reproduced below.

ARTICLE DETAILS

**TITLE (PROVISIONAL)**	Mechanistic Role of Long Non-Coding RNAs in the Pathogenesis of Metabolic Dysfunction-Associated Steatotic Liver Disease and Fibrosis
**AUTHORS**	Wade, Henry; Pan, Kaichao; Zhang, Bingrui; Zheng, Wenhua; Su, Qiaozhu

VERSION 1 - REVIEW

**REVIEWER NAME**	Zhou, Huiping
**REVIEWER AFFILIATION**	Virginia Commonwealth University
**REVIEWER CONFLICT OF INTEREST**	NA
**DATE REVIEW RETURNED**	27-Aug-2024

**GENERAL COMMENTS**	Overall, the manuscript provides a thorough and well-organized review of the current state of knowledge regarding lncRNAs in MASLD and liver fibrosis.<br>There are a few minor comments.<br>• In “2. Biogenesis and characteristics of Long-non-coding RNAs”, it will be helpful to clarify their unique roles for readers who may be less familiar with RNA biology, such as chromatin remodeling, scaffolding of protein complexes, and direct modulation of transcriptional activity. Adding a brief note on the challenges of studying lncRNAs, such as their tissue-specific expression and limited conservation across species, could provide context for the following discussions on therapeutic potential.<br>• In section 3, Consider dividing the content into subsections based on specific cell types or pathways (e.g., lipid metabolism, fibrogenesis, inflammation). This will improve readability and help guide the reader through the complex information.<br>• The section 4 provides important context regarding the challenges and ongoing efforts in translating lncRNA research into therapeutic applications. However, there are opportunities to improve clarity, expand on key points, and enhance the connection between lncRNAs and their therapeutic potential in MASLD and liver fibrosis.

**REVIEWER NAME**	Yang, Zhihong
**REVIEWER AFFILIATION**	Indiana University School of Medicine
**REVIEWER CONFLICT OF INTEREST**	none
**DATE REVIEW RETURNED**	02-Sep-2024

**GENERAL COMMENTS**	Metabolic dysfunction-associated steatotic liver disease (MASLD), previously referred to as nonalcoholic fatty liver disease (NAFLD), encompasses a broad range of hepatic metabolic disorders. And it may progress to metabolic dysfunction-associated steatohepatitis (MASH) and fibrosis, eventually develop to hepatocellular carcinoma (HCC). Dysregulation of long non-coding RNAs (lncRNAs) has been implicated in the development and progression of MASLD, involving multiple cell types in the liver. In current manuscript, the authors overview recent findings on the potential role of lncRNAs in the pathogenesis of MASLD and liver fibrosis via modulation of multiple pathways in different liver cells. This review will provide valuable update information related to lncRNAs and MASLD, which could benefit the research in studying liver disease. To further improve the manuscript, the following comments were provided:<br>1. Figure legend should be prepared or provided in more details. Please remove the box for “Downregulated” and “Upregulated”, it can be described in figure legend with some sentences like “lncRNA in red box was downregulated in MASLD, while LncRNAs in blue boxes were upregulated in MASLD”.<br>2. Please provide full name of “HC” since it is easy to be confused with other term, like Healthy Control (HC). As well as other cell type names: HSC, KC.<br>3. In Figure 1, “B-oxidation” should be “β-oxidation”. “B-catenin” should be “β-catenin”<br>4. Both sides of the word of “Ubiqutination” were lost in Figure 1. “Inflammation” in Figure 2;<br>5. In Figure 2, Please use full name of HH (Hedgehog).<br>6. In page 19 line 6, any words missed before “ inflammasome”?<br>7. How is lncRNAs in cholangiocyte and regulating bile acid signaling pathway in MASLD?<br>8. How is the diagnostic potential of lncRNAs in clinic?

VERSION 1 – AUTHOR RESPONSE

1. <u>Reviewer-1</u>

Comments to the Author

Overall, the manuscript provides a thorough and well-organized review of the current state of knowledge regarding lncRNAs in MASLD and liver fibrosis.

Response: We would like to thank the reviewer for the positive comments on our manuscript.

There are a few minor comments.

In “2. Biogenesis and characteristics of Long-non-coding RNAs”, it will be helpful to clarify their unique roles for readers who may be less familiar with RNA biology, such as chromatin remodelling, scaffolding of protein complexes, and direct modulation of transcriptional activity. Adding a brief note on the challenges of studying lncRNAs, such as their tissue-specific expression and limited conservation across species, could provide context for the following discussions on therapeutic potential.

Response: An additional subsection has been added (section 2.1) to provide further insight into the mechanism of lncRNA function.

2. In section 3, Consider dividing the content into subsections based on specific cell types or

pathways (e.g., lipid metabolism, fibrogenesis, inflammation). This will improve readability and help guide the reader through the complex information.

Response: We thank the reviewer for the constructive comments. The lncRNAs discussed in our manuscript have been presented under subsections of 3 major hepatic cell types, hepatocytes, hepatic stellate cells and Kupffer cells. Given to the limited availability of literature reports on some lncRNAs and the involvement of multiple pathways of other lncRNAs, we encountered difficulty to further subsection the information under each specific cell type.

3. The section 4 provides important context regarding the challenges and ongoing efforts in translating lncRNA research into therapeutic applications. However, there are opportunities to improve clarity, expand on key points, and enhance the connection between lncRNAs and their therapeutic potential in MASLD and liver fibrosis.

Response: Further information has been added to section 4.

2. Reviewer-2

Comments to the Author
Metabolic dysfunction-associated steatotic liver disease (MASLD), previously referred to as nonalcoholic fatty liver disease (NAFLD), encompasses a broad range of hepatic metabolic disorders. And it may progress to metabolic dysfunction-associated steatohepatitis (MASH) and fibrosis, eventually develop to hepatocellular carcinoma (HCC). Dysregulation of long non-coding RNAs (lncRNAs) has been implicated in the development and progression of MASLD, involving multiple cell types in the liver. In current manuscript, the authors overview recent findings on the potential role of lncRNAs in the pathogenesis of MASLD and liver fibrosis via modulation of multiple pathways in different liver cells. This review will provide valuable update information related to lncRNAs and MASLD, which could benefit the research in studying liver disease.

Response: We would like to thank the reviewer for the available comments on our manuscript.

To further improve the manuscript, the following comments were provided:

1. Figure legend should be prepared or provided in more details. Please remove the box for "Downregulated" and "Upregulated", it can be described in figure legend with some sentences like "lncRNA in red box was downregulated in MASLD, while LncRNAs in blue boxes were upregulated in MASLD".

Response: Figure legends have been amended as recommended.

2. Please provide full name of "HC" since it is easy to be confused with other term, like Healthy Control (HC). As well as other cell type names: HSC, KC.

Response: We have amended the text to provide the full name of these cells throughout.

3. In Figure 1, "B-oxidation" should be "b-oxidation". "B-catenin" should be "b-catenin"

Response: This has been corrected.

4. Both sides of the word of "Ubiqutination" were lost in Figure 1. "Inflammation" in Figure 2;

Response: This has been corrected.

5. In Figure 2, Please use full name of HH (Hedgehog).

Response: This has been corrected.

6. In page 19 line 6, any words missed before “inflammasome”?

Response: The text has been amended to complete the sentence.

7. How is lncRNAs in cholangiocyte and regulating bile acid signaling pathway in MASLD?

Response: We thank the reviewer for raising this question. A couple lncRNAs (i.e., ACTA2-AS1 and SNHG3) have been reported to be associated with the development of cholangiocarcinoma and ductular reaction. Unfortunately, due to the theme and limit space of this manuscript, this information was not included in this manuscript.

8. How is the diagnostic potential of lncRNAs in clinic?

Response: At the present time, utilization of lncRNAs as biomarkers for clinical diagnosis of MASLD has been limited due to the relatively low sample sizes of preclinical studies and limited reports on this topic. A paragraph has been added to the end of section 4 to address the reviewer’s comments.

3. Associate Editor

Comments to the Author: Both reviewers agreed this review manuscript covered important topics timely, but some clarifications are needed to improve the quality.

We thank the associate editor’s positive comments on our manuscript

VERSION 2 - REVIEW

**REVIEWER NAME**	Zhou, Huiping
**REVIEWER AFFILIATION**	Virginia Commonwealth University
**REVIEWER CONFLICT OF INTEREST**	No
**DATE REVIEW RETURNED**	03-Oct-2024

**GENERAL COMMENTS**	Well done

**REVIEWER NAME**	Yang, Zhihong
**REVIEWER AFFILIATION**	Indiana University School of Medicine
**REVIEWER CONFLICT OF INTEREST**	No
**DATE REVIEW RETURNED**	01-Oct-2024

**GENERAL COMMENTS**	All questions were answered.
